# The p53-caspase-2 axis in the cell cycle and DNA damage response

**DOI:** 10.1038/s12276-021-00590-2

**Published:** 2021-04-14

**Authors:** Yoon Lim, Loretta Dorstyn, Sharad Kumar

**Affiliations:** Centre for Cancer Biology, University of South Australia, GPO Box 2471, Adelaide, SA 5001 Australia

**Keywords:** Apoptosis, Experimental models of disease

## Abstract

Caspase-2 was discovered almost three decades ago. It was one of the first two mammalian homologs of CED-3, the other being interleukin 1β-converting enzyme (ICE/caspase-1). Despite high similarity with CED-3 and its fly and mammalian counterparts (DRONC and caspase-9, respectively), the function of caspase-2 in apoptosis has remained enigmatic. A number of recent studies suggest that caspase-2 plays an important role in the regulation of p53 in response to cellular stress and DNA damage to prevent the proliferation and accumulation of damaged or aberrant cells. Here, we review these recent observations and their implications in caspase-2-mediated cellular death, senescence, and tumor suppression.

## Cancer: Enzyme stabilizes tumor suppressor protein

Under conditions such as DNA damage or mitotic stress, the enzyme caspase-2 plays a vital role in regulating the stability of p53, a tumor suppressor protein. Caspases play multiple roles in cellular homeostasis, inflammation and programmed cell death. Sharad Kumar and colleagues at the Centre for Cancer Biology, UniSA in Adelaide, Australia, review current understanding of caspase-2 and its links with p53. Caspase-2 deficiency leads to defective p53 signaling, suggesting a regulatory link between the two molecules. Recent studies suggest that following DNA damage or cytokinesis failure caspase-2 cleaves MDM2, the p53 ubiquitin ligase, to stabilize p53. This prevents the proliferation of damaged cells, aneuploidy and accumulation of potentially premalignant cells. Reduced caspase-2 levels are associated with enhanced cancer progression in several models. The p53 regulatory function may explains this tumor suppressive activity of caspase-2.

## Introduction

Cysteine aspartate-specific proteases (caspases) are key drivers of inflammation and cell death. They mediate the processing and activation of proinflammatory cytokines or cleave several proteins during apoptosis to facilitate the programmed disassembly of cells^1,2^. Caspases also function in other pathways, including cell proliferation and differentiation. In general, caspases can be functionally split into two main groups. For example, mammalian caspase-2, -3, -6, -7, -8, -9, and -10 are apoptotic caspases, whereas caspase-1, -4, -5, -11, and -12 are involved in inflammation (or inflammation-induced cell death)^1,2^. In contrast to the other caspases, caspase-14 is mostly expressed and activated in the differentiating and cornifying layers of the epidermis and plays a crucial role in epidermal barrier formation^2^. The caspases implicated in apoptotic cell death can be further divided into initiators and effectors. As the term implies, initiator caspases are activated first in response to apoptotic signals, and once activated, they mediate the activation of effector caspases through proteolytic cleavage events. Effector caspases then cleave several hundred potential targets in the cell undergoing apoptotic dismantling. Initiator caspases are autoactivated^2,3^. They contain long prodomains near the N-terminus that comprise either death effector domains (DEDs; caspase-8 and -10) or caspase-recruitment domains (CARDs; caspase-2, -9, -1, and -11)^2,4^. These domains mediate protein dimerization and/or recruitment into larger complexes, such as apoptosomes^5^ and death-inducing signaling complexes (DISCs), to facilitate their activation^6^. The main effector caspases, caspases-3 and -7, do not possess the ability to autoactivate and their processing must be induced by initiator caspases^2^.

**Fig. 1:**
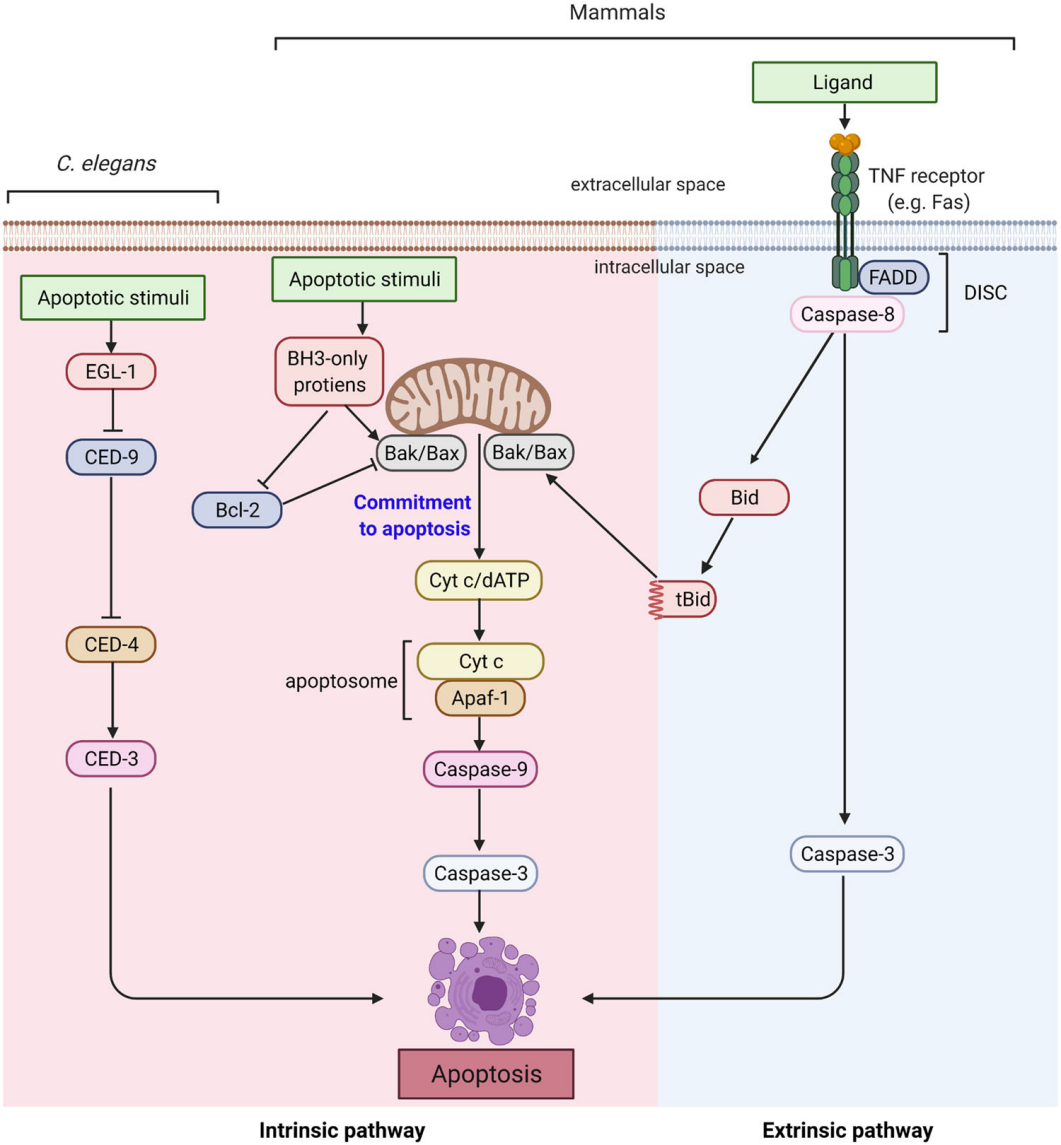
Conserved apoptotic pathways. Core apoptotic machinery in *C. elegans* and in mammals that drives caspase activation is shown. In mammals, there are two main apoptosis pathways. In the intrinsic pathway, various cellular insults result in MOMP and cytochrome c release from mitochondria, which drives apoptosome-dependent caspase-3 activation. In the extrinsic pathway, TNF family members activate caspase-8 via DISC. Activated caspase-8 and caspase-9 mediate the processing and activation of effector caspases, of which caspase-3 is the most prominent. The figure was created with BioRender.com.

Apoptotic caspase activation occurs via two main pathways. The mitochondrial pathway (BCL-2-regulated or the intrinsic pathway) is activated in response to various forms of cellular stress, including DNA damage, whereas the tumor necrosis factor (TNF) family of ligands initiates the alternative (extrinsic) pathway of apoptosis by binding to their cognate receptors (Fig. 1)^1,3,6^. The mitochondrial pathway involves the proapoptotic BCL-2 family members BAX and BAK^7^, which induce mitochondrial outer membrane permeabilization (MOMP)^8,9^ and promote cytochrome c release. The apoptotic protease-activating factor-1 (APAF-1) associates with cytochrome c in a multimeric apoptosome to activate caspase-9^5^. In the extrinsic pathway, apoptosis signaling via TNF family members is initiated following ligand-dependent activation of TNF family death receptors (such as FAS, TNFR, TRAILR1, and TRAILR2)^6^. The cytoplasmic domains of active multimeric receptors recruit and activate caspase-8 or -10 via DISC, which consists of FAS-associated death domain protein (FADD) and/or TNFR-associated death domain protein (TRADD), as well as other proteins (e.g., TRAFs and RIP1) that modulate death receptor signaling^10^. Activated caspase-8 can activate caspase-3 and caspase-7^11^ and cleave BID to a truncated form (tBID), which engages the mitochondrial pathway to amplify the apoptotic response through MOMP^12^ (Fig. 1).

**Fig. 2:**
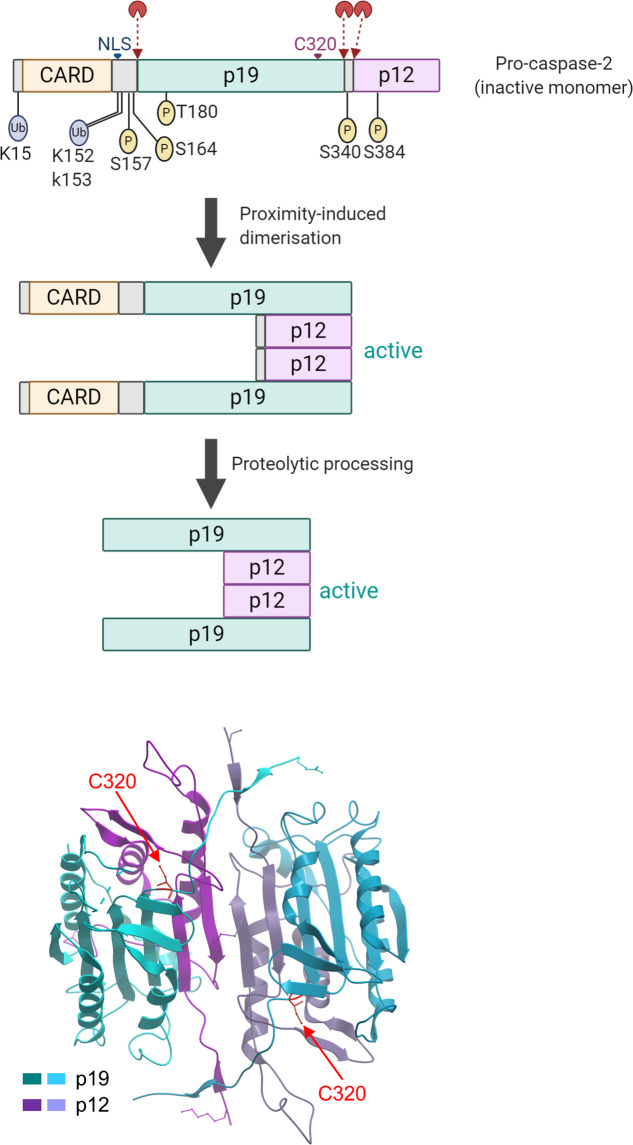
Caspase-2 structure and activation. In the primary structure, the location of zymogen cleavage sites, CARD, p19, p12, nuclear localization signal (NLS), and the catalytic Cys residue are shown. The putative ubiquitination and phosphorylation sites are also indicated. Various biochemical steps that lead to mature dimeric caspase-2 are shown. A ribbon diagram displays the dimeric structure of caspase-2 with two substrate binding pockets, including catalytic Cys (PDB ID: 3R6G)^32^. The figure was created with BioRender.com.

The focus of this review is caspase-2, a CARD-containing caspase. It is the most evolutionarily conserved member of the caspase family, with 31% identity (55% similarity) with the *C. elegans* caspase CED-3 and 25% identity (40% similarity) with the only *Drosophila* initiator caspase: DRONC^13–15^. Caspase-2 is expressed in almost all metazoan cells and is rapidly processed in response to various intrinsic and extrinsic apoptotic signals^3,13,16–23^. Similar to other initiator caspases, caspase-2 activation occurs via CARD-mediated homodimerization and autoprocessing^24–27^ (Fig. 2). Caspase-2 is also recruited to a large multiprotein complex called the PIDDosome^28^ (see below). Caspase-2 is the only caspase with a classical nuclear localization signal that mediates its predominant localization to the nucleus^29^. While caspase-2 is implicated in many cell death and non-cell death functions^30^, here, we discuss recent data that link caspase-2 to p53 stability and its various functions, including cell cycle regulation and tumor suppression.

## Caspase-2 activation

Through its CARD, overexpressed caspase-2 can rapidly oligomerize, which is sufficient for its activation^24,26,29^. Caspase-2, a zymogen with basal enzyme activity that is promoted by dimerization, is further activated by its proteolytic processing into two subunits, p19 and p12^31^ (Fig. 2). Structural studies suggest that mature caspase-2 forms a p19/p12 homodimer in solution with two active sites^32^. Biochemical data suggest that caspase-2 can be initially activated without processing and without other proteins^24^; however, in cell extracts, caspase-2 is recruited to a large complex, which probably mediates its full activation in response to various signals^25^. One such activation complex is the PIDDosome, which comprises PIDD1 (p53-induced death domain-containing protein 1), RAIDD (RIP-associated ICH-1/CED-3 homologous proteins with a death domain), and caspase-2^33,34^. The PIDDosome is considered to be the main signaling platform for caspase-2 activation in response to DNA damage^35,36^ and cytokinesis failure^37^. Somewhat surprisingly, however, PIDD1- or RAIDD-deficient cells and mice show normal caspase-2 activation^38–40^. Caspase-2 can also be activated in response to bacterial toxins in a PIDDosome-independent manner^41^.

The serine/threonine kinase ATM (ataxia telangiectasia mutated) has been shown to directly promote PIDDosome formation induced by DNA damage^35,38,39^. ATM phosphorylates Thr788 in the death domain (DD) of PIDD1, leading to conformational changes and allowing binding with RAIDD through DD interactions^35,42^. More recently, the phosphoprotein nucleophosmin (NPM1) has been shown to control PIDDosome formation specifically in the nucleolus, which activates caspase-2 in response to DNA damage^43^. The authors also demonstrated a RAIDD-dependent, but PIDD1-independent, platform for caspase-2 activation in the cytoplasm, while both RAIDD and PIDD1 are involved in a nucleolar platform^43^.

Although many studies have demonstrated the physiological importance of PIDDosome complex as a caspase-2 activation platform, other potential mechanisms of caspase-2 activation have also been reported. As mentioned above, caspase-2 was also shown to be activated in DISC, which includes CD95 (FAS/APO-1) and TNFR1^44,45^. Recently, NLRP3 has also been proposed to be a possible caspase-2 activation platform^46^. In this study, NLRP3 was translocated to mitochondria upon ER stress, leading to caspase-2 activation following the release of mitochondrial DNA and cytochrome c, resulting in inflammasome activation^46^. In addition, TRAF2 was shown to promote caspase-2 activation in a complex following cisplatin treatment of cells^47^. PIDD1 and RAIDD were not essential in this context^47^.

Caspase-2 activity can be regulated by several factors, including its localization, transcription and posttranslational modifications, including phosphorylation and ubiquitination^47,48^. A recent study reported that caspase-2 ubiquitination is required to stabilize caspase-2 dimerization and activation in a TRAF2-dependent manner in cultured cells^47^. Specifically, caspase-2 dimerization and further activation upon cisplatin treatment involved TRAF2-mediated ubiquitination at Lys15, Lys152, and Lys153^47^.

To date, several phosphorylation sites have been found to be important for caspase-2 regulation in certain contexts. Under nutrient-replete conditions that satisfy the pentose phosphate pathway in *Xenopus* oocytes, Ser135 (Ser164 in humans) of caspase-2 is phosphorylated by calcium/calmodulin-dependent kinase II (CAMKII), resulting in inhibition of caspase-2 activation^49^. Caspase-2 activation is also inhibited by phosphorylation at Ser157 by the protein kinase casein kinase-2 (CK2) in TNF-alpha-related apoptosis-inducing ligand (TRAIL)-mediated apoptosis^50^. A recent study showed that phosphorylation at Thr180 of caspase-2 is p38 mitogen-activated protein kinase (MAPK)-dependent and important in sterol regulatory element-binding protein 2 (SREBP2) regulation in lipid metabolism^51^. It is unclear whether p38 MAPK phosphorylates Thr180 directly. In the context of the cell cycle and DNA damage response, phosphorylation-dependent control of caspase-2 activity may be most relevant. For example, CDK1-Cyclin B1-dependent phosphorylation at Ser340 inhibits caspase-2 to protect cells from incidental apoptotic death during mitosis^52^. Recent data indicate that a key mitotic kinase, Aurora kinase B (AURKB), phosphorylates caspase-2 at the highly conserved residue Ser384^53^. A phosphomimetic mutation at Ser384 blocks caspase-2 catalytic activity, preventing the cleavage of its substrates, BID and MDM2, in cells with mitotic errors. Structural analysis suggests that phosphorylation at Ser384 may prevent substrate binding^53^. Therefore, phosphorylation prevents cleavage of the key caspase-2 substrates involved in apoptosis and p53 stabilization (see below).

## Caspase-2 – cell cycle connection

Possible links between caspase-2, p53 and the cell cycle have been consistently reported. For example, caspase-2-deficient murine embryonic fibroblasts (MEFs) proliferate faster and become immortalized quicker than their wild-type counterparts^54,55^. Caspase-2 has been shown to interact with cyclin D3, which regulates the G1/S transition during the cell cycle^56^. Furthermore, as stated above, caspase-2 can be phosphorylated by CDK1-Cyclin B1, a mitosis-promoting kinase, during mitosis^52^. This phosphorylation inhibits caspase-2 activation, preventing unwanted cell death during mitosis. A role for caspase-2 in mitotic catastrophe was proposed approximately 15 years ago^57^. Mitotic catastrophe is programmed cell death resulting from failed mitosis, including delayed mitosis and failure to repair DNA damaged by physical or chemical stresses^1,58^. CHK2 inhibition was shown to induce mitotic catastrophe and caspase-2-dependent cell death, and the inhibition/ablation of caspase-2 suppresses mitotic catastrophe, resulting in multinucleated and aneuploid cells^57^. Later, Sidi and colleagues showed that inhibition of CHK1, a cell cycle regulating kinase, causes ATM/ATR-mediated caspase-2 activation and results in nonapoptotic cell death program initiation, even in p53-deficient cells^59^. These reports suggest that inhibition of either CHK1 or CHK2 results in mitotic catastrophe involving caspase-2-mediated cell death.

Ongoing errors in chromosome segregation caused by dysfunctional cell cycle checkpoints, such as spindle assembly checkpoints (SACs) or cytokinesis failure during mitosis, can cause aneuploidy, which often leads to chromosomal instability (CIN), a hallmark of cancer^60^. This failure is often associated with defects in the activation of apoptotic pathways. For example, mutation of the p53 tumor suppressor or overexpression of the prosurvival BCL-2 family of proteins are common precursors to malignant transformation and therapy resistance^61,62^.

**Fig. 3:**
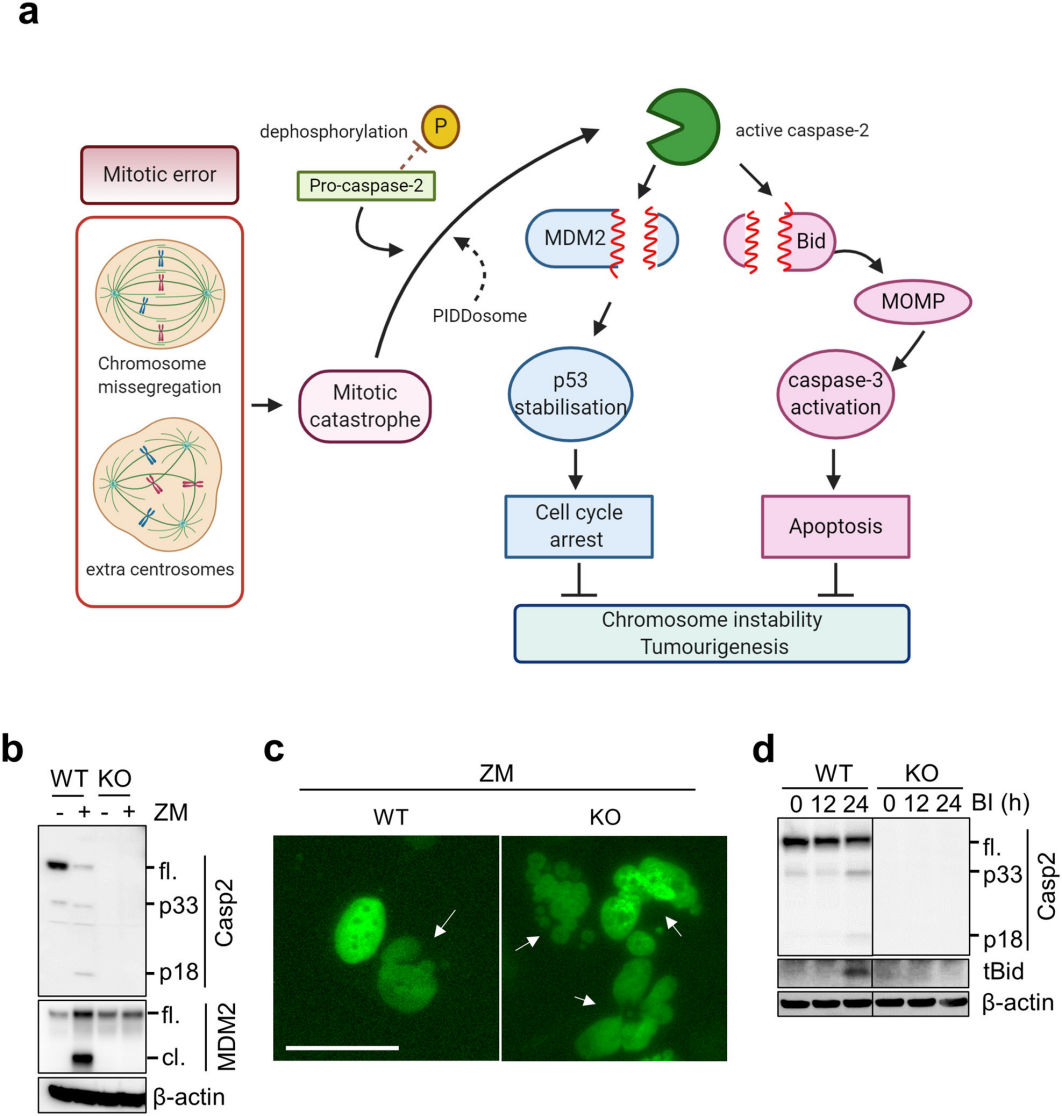
Caspase-2 functions in cell cycle arrest and apoptosis. **a** Schematic showing that the cell cycle arrest and apoptotic functions of caspase are mediated via cleavage of MDM2 and BID following mitotic catastrophe, respectively. Figure created with BioRender.com. **b** An example of MDM2 cleavage by caspase-2 (Casp2) following treatment of WT or caspase-2-deficient (KO) U2OS cells with or without the AURKB inhibitor ZM447439 (ZM). **c** Panels showing examples of increased multinucleated (arrow) caspase-2-deficient U2OS cells following AURKB inhibition. Scale bar, 50 µm. **d** Examples of caspase-2-mediated BID cleavage (appearance of truncated BID, tBID) following treatment with a PLK1 inhibitor, BI2536 (BI), which induces mitotic arrest. **fl**.; full-length, **cl**.; cleaved, **p33** and **p18**; cleaved caspase-2. The data and images in **b**–**d** were generated by Yoon Lim.

Caspase-2 deficiency is closely associated with enhanced aneuploidy in primary MEFs and tumors^13,54,63–65^. Similarly, bone marrow cells from aged but not young caspase-2*-*deficient mice also showed significantly higher aneuploidy than bone marrow cells from WT mice^66^. Recent studies showed that increased aneuploidy in caspase-2-deficient and caspase-2-catalytic mutant mouse cells reduced BID cleavage and cell death and clonogenic survival following polo-like kinase 1 (PLK1) inhibition in caspase-2-deficient cells (Fig. 3a, d)^67^. This finding implies a requirement for the enzymatic activity of caspase-2 in the apoptosis of aneuploid cells; however, it is unknown whether the PIDDosome is essential.

BUBR1 is a core protein of the mitotic checkpoint complex in mitosis control, and it has been shown to be an inhibitor of the PIDDosome in response to irradiation-mediated DNA damage^36^. Specifically, following DNA damage during mitosis, BUBR1 recruits ATM phosphorylated PIDD1 to the kinetochore, outcompeting RAIDD for PIDD1 binding, thus preventing PIDDosome formation. This mechanism helps prevent unplanned cell death during cell division and implicates the PIDDosome and caspase-2 as critical mitosis regulators^36^.

## Caspase-2 stabilizes p53 following DNA damage

In addition to the findings discussed above, caspase-2 deficiency has often been linked to a defective DNA damage response. For example, caspase-2-deficient MEFs readily escape senescence in culture and exhibit increased micronuclei formation and sustained DNA damage during cell culture and following *γ*-irradiation^54^. A lack of caspase-2 is known to be associated with increased aneuploidy in both MEFs and in E*μMyc* lymphoma cells, and loss of caspase-2 leads to defective p53-mediated signaling, suggesting that caspase-2 is important in maintaining genomic integrity during cell proliferation and following DNA damage^54,55^. Other studies have implicated both PIDD1 and caspase-2 in DNA damage-dependent p53-mediated apoptosis^68,69^. Recent observations indicate that active caspase-2 stabilizes p53, resulting in cell cycle arrest and/or apoptosis (Fig. 3). The levels of p53 are regulated by the ubiquitin ligase MDM2, which is critical for the ubiquitination and degradation of p53^70–72^. In a seminal study published in 2011, Oliver and colleagues found that caspase-2 directly cleaves MDM2 at Asp367, resulting in the removal of the C-terminal RING domain that is critical for p53 ubiquitination^68^. N-terminally truncated MDM2 then binds p53 and promotes its stability^68^. Thus, this finding suggested for the first time that, following genotoxic stress and DNA damage, p53-dependent induction of the PIDDosome and caspase-2 activation generates a positive feedback loop that inactivates MDM2 and promotes p53 stability.

**Fig. 4:**
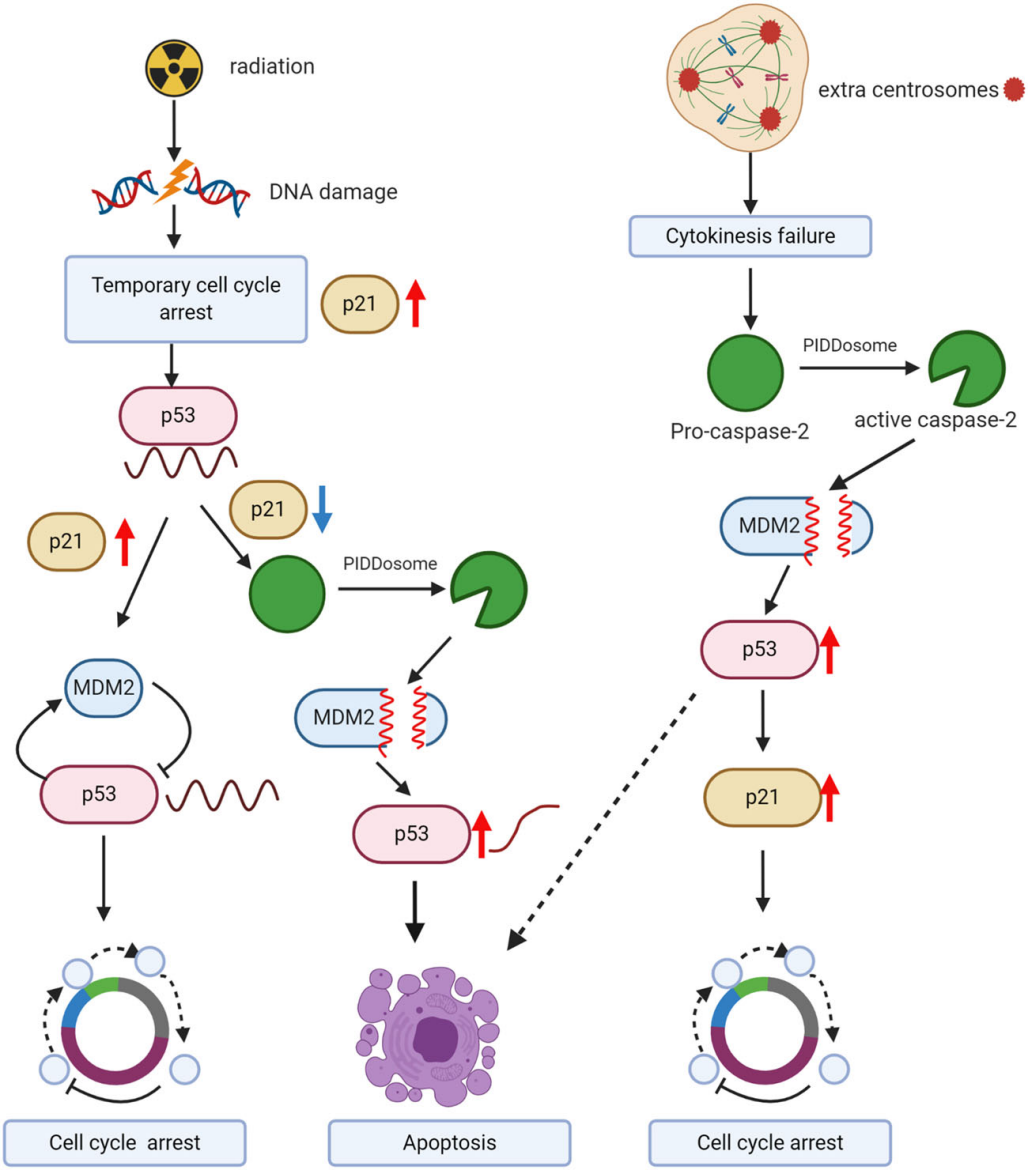
Different physiological outcomes mediated by caspase-2-dependent p53 stabilization. A proposed scheme showing that PIDDosome-mediated caspase-2 activation, MDM2 cleavage and p53 stabilization serves as a checkpoint under conditions of sustained DNA damage to induce cell cycle arrest or apoptosis. The figure was created with BioRender.com.

As MDM2 is also a transcriptional target of p53, increased p53 levels drive MDM2 expression, which in turn results in p53 degradation via the ubiquitin-proteasome system^73,74^. Thus, an MDM2-p53 negative feedback loop results in oscillatory p53 pulses following double-stand DNA breaks and ATM activation^75–77^. On the other hand, in response to UV-induced DNA damage that activates ATR, p53 is expressed as a broad pulse^78^. A study published in 2020 suggests that several days after ionizing radiation-induced DNA damage, some cells switch from oscillating to sustained p53 dynamics^79^. Using a Venus fluorescent reporter to monitor p53 levels in single cells, the authors found that following 10 Gy irradiation, 15–20% of cells exhibit stably increased p53 levels^79^. Interestingly, the fraction of cells switching from pulsatile to sustained p53 levels was maximal at 5 Gy but reduced with increasing radiation dose of 10 or 20 Gy dose. The cells with sustained p53 signaling showed chromosomal damage. The authors found that late phase p53 levels were reduced in irradiated PIDD1-deficient cells and in cells following caspase-2 inhibition^79^. Overall, their data suggest that PIDD1 and caspase-2 are required for cells that evade DNA damage-induced cell cycle arrest to switch from pulsating to sustained p53 signaling. Thus, the presence of damaged DNA activates caspase-2 via the PIDDosome to stabilize p53 signaling and thus limits the proliferation of cells carrying extensive DNA damage by inducing apoptosis (Fig. 4).

## Caspase-2 in ploidy control

A study by Fava and coworkers demonstrated that extra centrosomes resulting from cytokinesis failure trigger PIDDosome-dependent caspase-2 activation^37^. Activated caspase-2 then cleaves MDM2, leading to p53 stabilization and p21-mediated cell cycle arrest of polyploid cells (Fig. 3a–c). The study showed that PIDD1 can localize to extra mother centrioles and that caspase-2 activation following AURKB inhibition is dependent on PIDD1 and RAIDD^37^. Importantly, the PIDDosome was essential for caspase-2 activation in this context and was required to protect cells against polyploidization and malignant transformation^37^. A more recent study showed p53 stabilization and effective cell cycle arrest following treatment with the same AURKB inhibitors in caspase-2-deficient U2OS cells^80^, suggesting that caspase-2-mediated p53 stabilization may be cell-type specific. Caspase-2 and PIDD1 have also been shown to be involved in the control of ploidy during postnatal development and regeneration in the liver^81^. Two very recent papers demonstrated how the PIDDosome may control centrosome amplification and aneuploidy^82,83^. The authors found that PIDD1 is directly recruited to mature centrosomes by the centriolar distal appendage protein ANKRD26, and this interaction is required for PIDDosome and caspase-2 activation following centrosome amplification^82,83^. These reports suggest that ANKRD26-dependent PIDDosome activation acts as a centriolar signal to limit cell proliferation via the caspase-2-p53 axis in response to centrosome amplification and extra chromosomes.

## Caspase-2 as a tumor suppressor

From the discussion above, it is apparent that caspase-2 plays an important checkpoint function by stabilizing p53 to prevent the accumulation of cells carrying damage, thus potentially acting to suppress tumorigenesis. Indeed, there are several lines of evidence to suggest that loss of caspase-2 is associated with increased susceptibility to certain types of tumors. The human *CASP2* gene is in Ch7q34^84^. As Ch7q is often deleted in hematological malignancies^85^, reduced expression of caspase-2 (along with other genes in this region) is not uncommon. Reduced caspase-2 expression has been reported in some cases of Burkitt lymphoma, mantle cell lymphoma (MCL), chronic lymphocytic leukemia (CLL), and acute myeloid leukemia (AML)^86,87^. In AML, reduced caspase-2 levels are linked to poor prognosis^88^. *CASP2* mutations are rare but sometimes associated with colon, gastric lung and breast cancer^89,90^. Interestingly, aneuploidy tolerance and chromosomal instability in colon cancer correlate with low *CASP2* levels due to *BCL9L* gene aberrations^91^.

In mouse models employing caspase-2 deficiency, a tumor suppressive effect of caspase-2 has been reported by several laboratories (Table 1). For example, caspase-2 deficiency enhances lymphomagenesis in *Eμ-Myc* transgenic mice^55^^,^^64,92^ and ATM kinase-deficient animals^63^ and accelerates breast tumor formation in *MMTV/c-neu* mice^65^ and in a *Kras*^*G12*^^*D*^*-*driven lung adenocarcinoma model^93^. The tumor suppressor role of caspase-2 was also apparent in a diethylnitrosamine-induced mouse hepatocellular carcinoma (HCC) model, where at 10 months of age, all caspase-2-knockout animals developed malignant HCC, whereas the WT animals showed only adenomas and rarely HCC^94^. Surprisingly, a new study showed that caspase-2 loss had no effect on diethylnitrosamine-induced carcinogenesis^95^. The reason for this discrepancy is unclear, but as the severity and timing of diethylnitrosamine-induced tumor appearance is dependent on many factors, the rate of HCC development may have masked the effects of caspase-2 deficiency. On the other hand, and interestingly, Sladky et al.^95^ found that PIDDosome deficiency caused hyperpolyploidization that prevented HCC development. In addition, caspase-2 deficiency somewhat delayed neuroblastoma development driven by the *TH*-MYCN transgene in mice^96^. Consistent with these findings, an analysis of expression array datasets of primary human neuroblastoma tumor patients demonstrated a significant correlation between high levels of caspase-2 and poor prognosis^96^. Notably, this correlation was observed in neuroblastoma patients with nonamplified MYCN^96^. The tissue- and context-specific tumor suppressor function of caspase-2 was also apparent in the distinct transcription profiles of caspase-2-deficient *Eμ-Myc* and *TH*-MYCN mouse tumors^97^.

**Table 1:** Mouse tumor models used to study the effects of caspase-2 deficiency on tumourigenesis.

Mouse model	Tumor	Effect of caspase-2 deficiency	Refs
*Eµ-Myc*	Lymphoma	Heterozygous and homozygous caspase-2 deficiency accelerated lymphoma development in *Eµ-Myc* mice.	^55,64,92^
*Atm*^*−/−*^	Thymoma	Accelerated thymic tumor development in *Atm*^−*/−*^ mice.	^63^
*MMTV/c-neu*	Mammary tumor	Homozygous caspase-2 deficiency accelerated mammary tumor formation in *MMTV/c-neu* mice.	^65^
*TH-MYCN*	Neuroblastoma	Heterozygous and homozygous caspase-2 deficiency delayed neuroblastoma development in *TH-MYCN* mice.	^96^
*Kras*	Lung tumor	Homozygous caspase-2 deficiency accelerated proliferation and progression of lung tumors driven by *Kras*.	^93^
Diethylnitrosamine	Hepatocellular carcinoma	Accelerated hepatocellular carcinoma development.	^94^
		Suppression of hepatocellular carcinoma development.	^95^

## Perspective

The work summarized here indicates that both the PIDDosome and caspase-2 play critical roles in p53 stabilization and cell death to prevent polyploidy, which is required to prevent the accumulation of harmful cells with chromosomal abnormalities. Importantly, caspase-2 activation can also occur independent of the PIDDosome to prevent aneuploidy and tumorigenesis. These data suggest that caspase-2 activation serves as a checkpoint under specific conditions of sustained cellular or DNA damage, which may explain the tumor suppressive effects of caspase-2 seen in various mouse models. However, many questions remain. Perhaps the most puzzling finding is that caspase-2-deficient mice acquire only mild phenotypes and do not spontaneously develop tumors or other specific pathologies in old age. Mice lacking caspase-2 demonstrate increased ploidy in the liver, and aged caspase-2 mice show increased aneuploidy in the bone marrow compartment^66^. Interestingly, this study also showed a possible role of caspase-2 in regulating hematopoietic stem cells (HSCs) and in progenitor cell differentiation^66^. Caspase-2-deficient animals showed a significant increase in short-term HSCs and multipotent progenitors with skewed differentiation towards myeloid progenitors with age^66^. Whether this phenotype represents a premyeloid state is unclear. Nevertheless, from studies with caspase-2-deficient mouse models, it is clear that caspase-2 deficiency is not a primary driver of tumorigenesis. Furthermore, it has been demonstrated that loss of caspase-2 significantly improves energy metabolism and reduces weight gain in mice on a high-fat diet that is associated with reduced incidence of non-alcoholic fatty liver disease (NAFLD) progression^98–100^. However, while caspase-2 has been shown to regulate both metabolism and polyploidization in the liver^81,98–100^, there appear to be no dramatic consequences in normal liver pathophysiology in caspase-2-deficient mice. Finally, caspase-2 deficiency is linked to aging-related traits, including increased oxidative stress and reduced levels of antioxidant signaling^101,102^. These findings support the idea that caspase-2 may play specific roles during replication stress or metabolic stress in cells. However, whether these phenotypes are acquired because of an alteration in caspase-2-mediated p53 regulation remains to be determined.
